# Key considerations for ELISA-based quantification of diverse amyloid beta forms in murine brain homogenates

**DOI:** 10.3389/fnins.2025.1645952

**Published:** 2025-10-01

**Authors:** Nicole G. Metzendorf, Dag Sehlin, Greta Hultqvist

**Affiliations:** ^1^Department of Pharmacy, Uppsala University, Uppsala, Sweden; ^2^Department of Public Health and Caring Sciences, Uppsala University, Uppsala, Sweden

**Keywords:** ELISA-based quantification, amyloid beta, Alzheimer’s disease, antibody, detection, aggregates, oligomers

## Abstract

Enzyme-Linked Immunosorbent Assay (ELISA) is a widely utilized method for quantifying amyloid beta (Aβ) levels in various biological samples, including brain homogenates. Aβ exist in multiple structural forms: monomers, soluble oligomers, protofibrils, and fibrils, each exhibiting distinct biochemical properties and degrees of neurotoxicity. Their toxic potential also varies by localization, whether intracellular, membrane-bound, or extracellular. Accurate detection and quantification of these diverse Aβ species and localizations are critical for understanding their roles in Alzheimer’s disease (AD) pathology. However, suboptimal ELISA configurations and misinterpretations of results can lead to misleading conclusions. This study highlights key considerations for optimizing ELISA protocols specifically for detecting distinct Aβ species and localizations, with a focus on applications in mouse brain tissue. We also provide guidance on antibody selection to improve selectivity and specificity of Aβ detection, ultimately enhancing the reliability and interpretability of ELISA-based Aβ measurements.

## 1 Introduction

In Alzheimer’s disease, the aggregation of amyloid beta (Aβ) plays a central role in disease progression and memory impairment. Aβ is generated through the cleavage of the amyloid precursor protein (APP), producing peptides of varying lengths, with Aβ40 and Aβ42 being the most common. In addition to Aβ40 and Aβ42, several other Aβ peptide lengths exist, each with distinct aggregation propensities and biological effects. Among these, Aβ42 is particularly prone to aggregation, and is strongly associated with the formation of toxic oligomers and amyloid plaques. The ratio between Aβ42 and Aβ40 is a critical factor influencing the overall likelihood of aggregation. Several familial Alzheimer’s disease-linked mutations, such as the Swedish, Flemish, and Austrian, affect both the cleavage efficiency of the amyloid precursor protein (APP) and the preferred length of the resulting Aβ peptides. Truncations of the N-terminal, such as Aβ3-40/42, are also common and contribute significantly to disease progression (Sims et al., 2023). Generally, higher concentrations of Aβ increase the risk of aggregation. Several factors can impede the clearance or degradation of Aβ, with age being one of the most prominent. Mutations such as the Arctic, Italian, and Dutch mutations (E22G, K and Q) make Aβ more prone to aggregation (Fawzi et al., 2008; Grant et al., 2007; Lord et al., 2009; Yang et al., 2023). Murine Aβ is less prone to aggregation compared to human Aβ, which is why mouse models of Aβ pathology express human APP or a humanized Aβ domain.

The aggregation cascade of Aβ begins when a single monomer misfolds, adopting a beta-hairpin structure that acts as a seed for the misfolding of additional monomers, which leads to the formation of oligomers. There are multiple definitions of Aβ oligomers used in the literature; throughout this manuscript, we define an Aβ oligomer as a small, soluble aggregate that contains the beta-hairpin structure (Larini and Shea, 2012; Ruttenberg and Nowick, 2024). Soluble Aβ oligomers are considered highly neurotoxic due to their ability to disrupt synaptic function, impair long-term potentiation, and induce neuronal dysfunction (Bode et al., 2019; Gallego Villarejo et al., 2022; Huang and Liu, 2020; Kayed and Lasagna-Reeve, 2013; Nguyen et al., 2022; Sengupta et al., 2016). Several clinical trials targeting insoluble Aβ aggregates (e.g., Aducanumab, Gantenerumab) have shown plaque clearance but limited or no cognitive benefit, suggesting that toxic effects arise earlier in the aggregation pathway and may be driven by soluble oligomers (Bateman et al., 2023; Sevigny et al., 2016; Tolar et al., 2021).

These oligomers vary in size and can remain soluble before assembling into larger structures known as protofibrils. Protofibrils are intermediates between oligomers and fibrils and are also associated with neurotoxicity, as they may represent a critical transition stage in Aβ aggregation. Certain therapeutic antibodies, such as lecanemab preferentially bind to soluble protofibrils and have demonstrated moderate slowing of cognitive und functional decline in clinical trials compared to placebo (Lannfelt et al., 2014; Ono and Tsuji, 2020; van Dyck et al., 2023). However, it remains unclear whether protofibrils maintain the beta-hairpin structure or undergo further conformational changes as they mature into fibrils.

As oligomers and protofibrils continue to grow, they gradually adopt more ordered beta-sheet conformation, marking structural transition from beta-hairpins to parallel beta-sheets (Sarroukh et al., 2010; Xu et al., 2005). This conformational shift results in the formation of fibrils, larger aggregates characterized by extensive beta-sheet content. While smaller fibrils can remain soluble, continued aggregation leads to the formation of insoluble fibrils that precipitate as extracellular plaques, one of the pathological hallmarks of Alzheimer’s disease.

Despite their abundance, plaques are generally considered less acutely toxic than earlier Aβ species such as oligomers or protofibrils. All antibodies that have shown clinical benefit, such as Aducanumab (targeting aggregated Aβ), Lecanemab (preferentially binding soluble protofibrils), and Donanemab (binding Aβ plaques via the pyroglutamate-modified N-terminus of Aβ42), also reduce plaque burden and a correlation between plaque clearance and cognitive benefit has been observed (Sevigny et al., 2016; Sims et al., 2023; van Dyck et al., 2023). However, these clinical benefits have been modest to moderate, typically reflecting a slowing of cognitive decline by approximately 20 – 30% compared to placebo. This highlights both the potential and the current limitations of targeting Aβ aggregates. Nevertheless, the precise contribution of individual Aβ species to disease progression has yet to be fully defined, underscoring the need for methods capable of distinguishing between monomers, oligomers, protofibrils, and fibrils in both basic and translational research.

**FIGURE 1:**
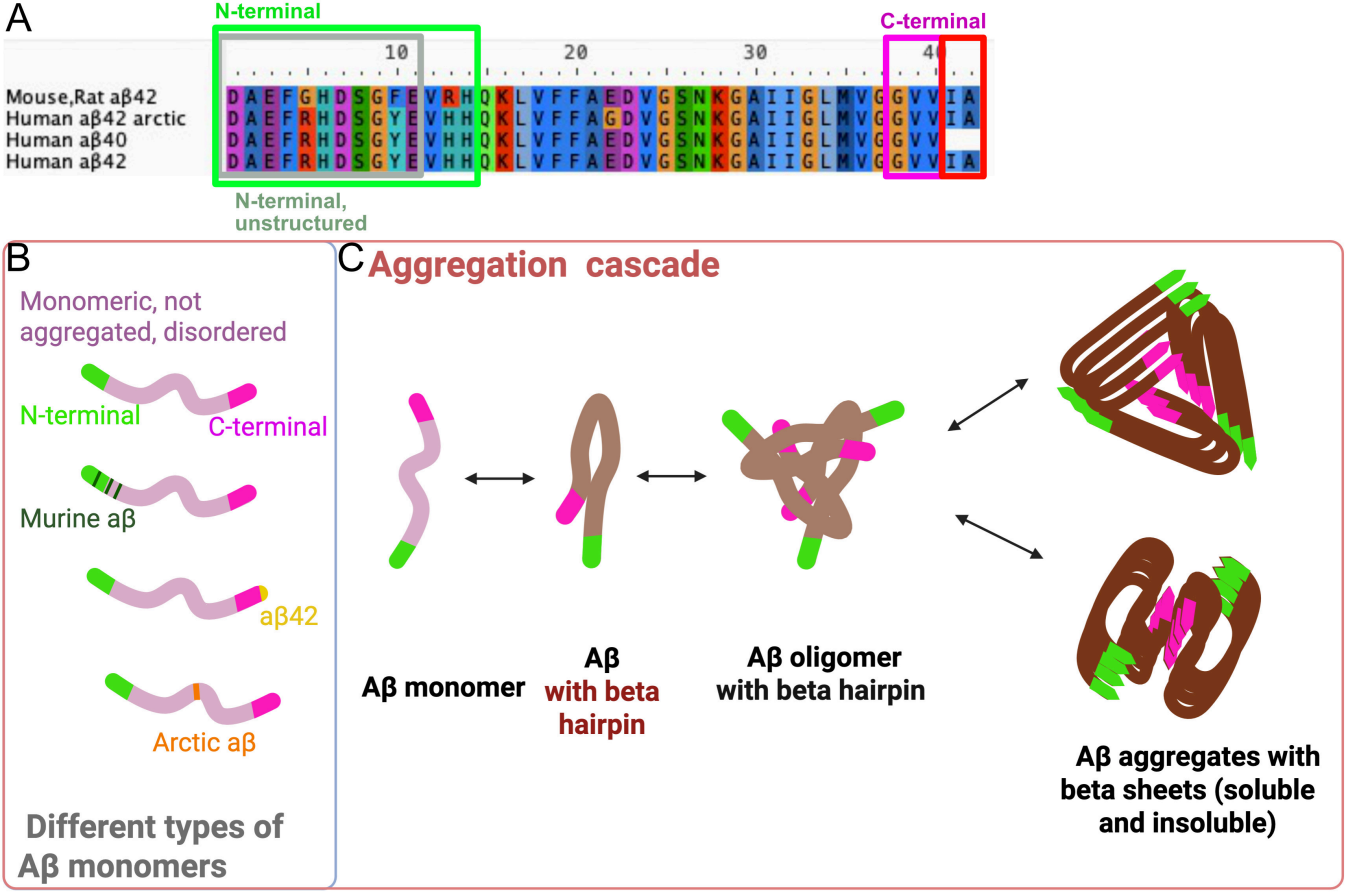
Different types of monomers and the Aβ aggregation cascade. **(A)** Sequence alignment of human and mouse/rat Aβ highlighting differences at amino acid (AA) position 5, 10 and 13. From AA 14 to 42, human and mouse Aβ are identical. The Arctic mutation, is for instance located at AA 22. The first 10–11 N-terminal AAs are unstructured, making it difficult to determine their structure in crystallization experiments, so they can only be studied in structures determined by NMR (see Figure 2). **(B)** There are different types of Aβ monomers, each with varying tendencies to aggregate. These monomers can differ in length (e.g., Aβ1-40, Aβ3-40 and Aβ1-42) and can contain mutations, such as the Arctic mutation, which enhances the aggregation properties. **(C)** Schematic overview of the Aβ aggregation cascade. Unstructured monomers misfold and begin to aggregate, initially forming small aggregates that adopt a beta-hairpin structure. As these aggregates grow larger, they twist into more ordered conformations, ultimately forming beta-sheets. Various aggregate folds have been identified (as seen in Figure 2), however, in most structures, the N-terminus remains more accessible for antibody binding, unless the N-terminus is truncated. In smaller aggregates, the C-terminus may also be available for binding. Created with Biorender.com.

A schematic diagram of this aggregation cascade, showing the stepwise transition from monomers to plaques, can be found in Figure 1.

**FIGURE 2:**
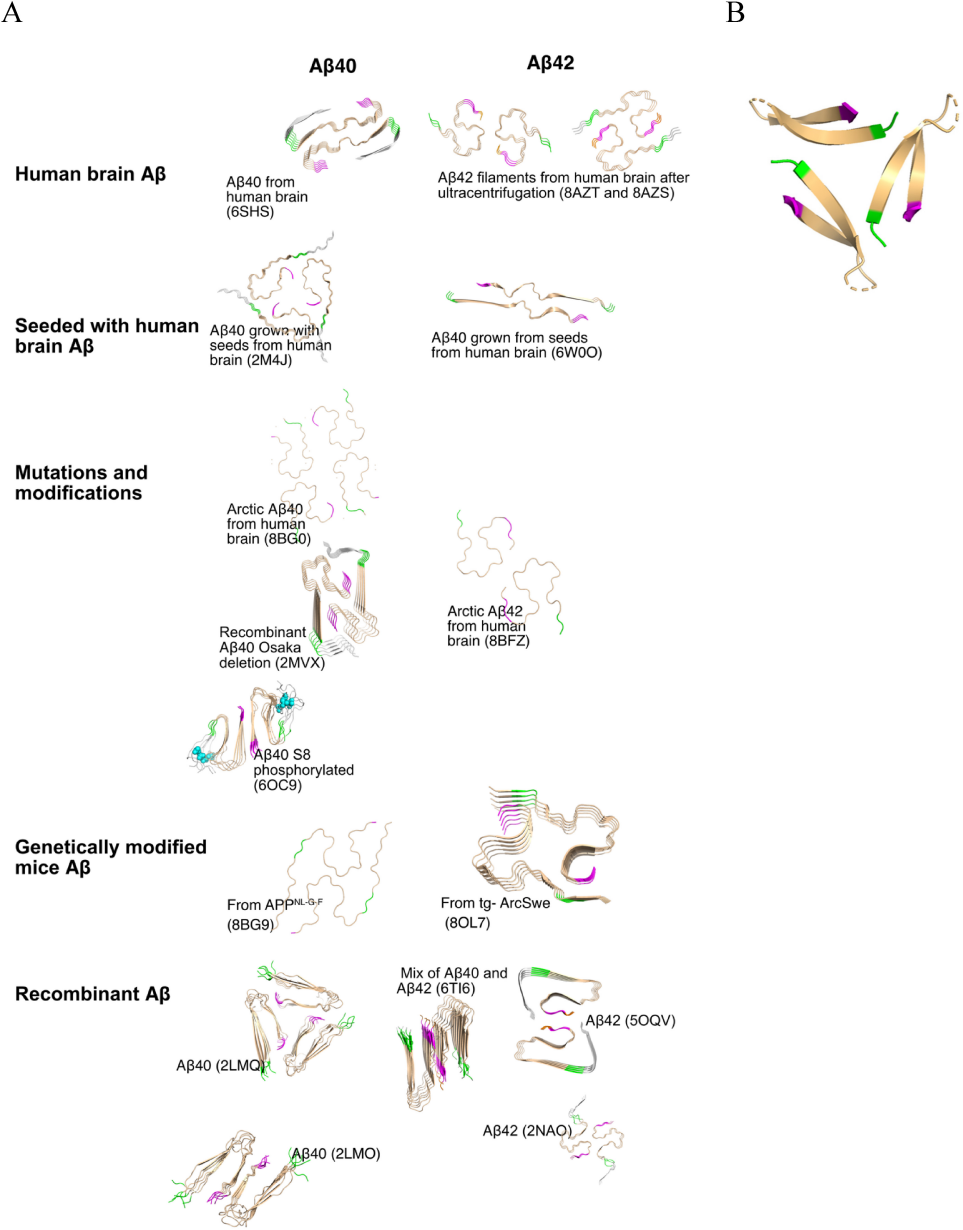
**(A)** Structures of Aβ fibrils with different origins. This panel displays the structures of Aβ fibrils derived from different origins, as indicated on the figure. The following PDB structures are shown: Human brain Aβ40 (6SHS), Human brain Aβ42 (8AZT), Aβ40 grown with seeds from human brain (2M4J), Aβ42 grown with seeds from human brain (6W0O), arctic Aβ40 from human brain (8BG0), recombinant Aβ40 with Osaka deletion (2MVX), arctic Aβ40 S8 phosphorylated (6OC9), arctic Aβ42 from human brain (8BFZ), Aβ40 from APP^NL–G–F^ mice (8BG9), Aβ42 from tg-ArcSwe mice (8OL7), recombinant Aβ40 (2LMQ and 2LMO), recombinant Aβ42 (5OQV and 2NAO) and mix of Aβ40 and Aβ42 (6Ti6). The N-terminal amino acids “VHH” are colored in green, and the C-terminal amino acids “GVV” are colored pink, as detailed in Figure 1A. **(B)** The structure of a stabilized Aβ oligomer containing a beta-hairpin conformation (PDB: 6cg5 visualized using PyMOL). The formation of such oligomers is an early step in the Aβ aggregation cascade. The beta-hairpin motif reflects a misfolded conformation that may serve as a seed for further aggregation. These oligomeric species play a critical role in the progression of Alzheimer’s disease, as they exhibit highly neurotoxicity and can significantly disrupt in cellular function (Xiao et al., 2015).

The oligomeric form of Aβ is widely considered to be the most toxic, potentially due to the hairpin structure and increased mobility, as highlighted in many studies (Celej et al., 2012; Lorenzen et al., 2014; Mrdenovic et al., 2022; Sandberg et al., 2010; Sengupta et al., 2016; Tolar et al., 2021). Understanding and quantifying various types of Aβ aggregates is crucial for improving our knowledge of different transgenic models, accurately characterizing disease types, and assessing the effects of various treatment strategies. Enzyme-Linked Immunosorbent Assay (ELISA) and other antibody-based methods are commonly used to quantify Aβ aggregates. However, designing an ELISA that accurately detects specific forms of Aβ is challenging. This is primarily due to the structural complexity of Aβ aggregates, where portions of the peptide (including the N- and C-terminal regions) may be hidden within the aggregate (Figure 2). Additionally, truncations at the N- and C-termini can alter the accessibility of epitopes, creating potential biases in detection. This structural heterogeneity can lead to misinterpretation of the results, as aggregates of different sizes and conformations may not be equally detected depending on the epitopes that are exposed.

To mitigate these challenges, it is essential to carefully select antibodies that target accessible epitopes specific to the aggregation state of interest. In some cases, using a combination of antibodies that recognize different regions of Aβ (e.g., N-terminal, C-terminal, or mid-region) may enhance the sensitivity and specificity of detection.

Equally important is the preparation of brain homogenates in a manner that enables the effective separation of soluble, membrane-bound, and insoluble Aβ species as the location of the aggregates also affects their toxicity (Gallego Villarejo et al., 2022). It is likely that the initial aggregation occurs inside the cells (Gao et al., 2021; Yu et al., 2018) and the aggregates associated with the cellular membrane are the most toxic (Mrdenovic et al., 2022; Peters et al., 2016). This allows for more accurate quantification of distinct Aβ species.

While ELISA remains a valuable tool, the inherent complexity of Aβ aggregation necessitates further optimization of assay setups to ensure more accurate detection of specific aggregate forms. Incorporating complementary techniques, such as mass spectrometry or FRET (fluorescence resonance energy transfer), could complement the read-outs from the ELISAs.

In this paper, we will outline effective methods for preparing tissue homogenate samples from Alzheimer’s disease mouse models for ELISA. We will focus on the composition and structure of various types of Aβ aggregates, with an emphasis on selecting the most appropriate antibodies for both coating and detection in an Aβ ELISA. Additionally, we will provide useful tips and tricks for optimizing ELISA setups to improve specificity in detecting different Aβ aggregate forms and cellular locations.

## 2 Materials and equipment

### 2.1 Key considerations for analyzing Aβ in homogenates using ELISA

#### 2.1.1 Homogenization and dilution

##### 2.1.1.1 Buffer additives

Although any tissue can be analyzed, the brain is the most relevant organ in Alzheimer’s disease studies due to its primary involvement in the disease. When homogenizing tissue, the choice of buffer is crucial. A buffer without detergent will fail to dissolve membranes, meaning that the supernatant after centrifugation will mainly contain soluble Aβ, excluding membrane-associated Aβ or Aβ within organelles, as the membranes are likely left intact during homogenization. The homogenization process may also disrupt some cells and break larger fibrils, potentially resulting in the detection of cytosolic proteins and fragments of fibrils in this fraction.

A buffer containing detergent, such as Triton-X100, will dissolve membranes and smaller organelles, allowing for the detection of Aβ bound to membranes, membrane proteins, and contents from inside the organelles. However, insoluble Aβ fibrils, like those found in plaques, will not be effectively solubilized by Triton-X100 (McDonald et al., 2012). While less stable protein interactions may be disrupted, these fibrils remain intact. To analyze insoluble Aβ fibrils, a stronger acid, such as formic acid (FA) or Guanidinium hydrochloride (GuHCl), is required to dissolve these aggregates into individual monomers, which can then be detected by ELISA (after neutralization).

**FIGURE 3:**
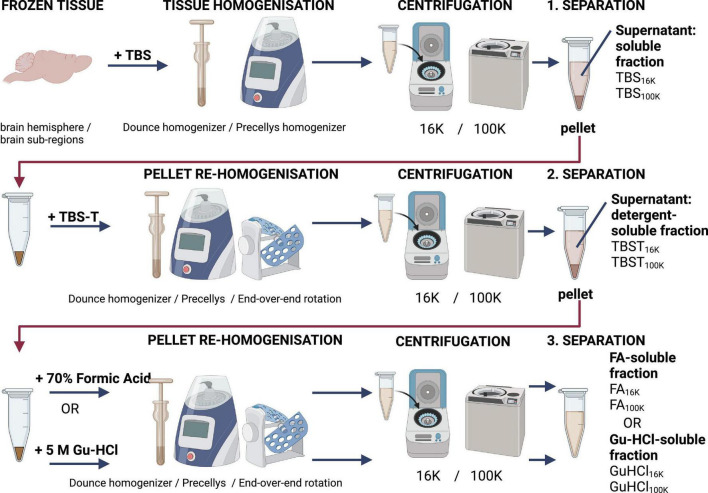
Schematic overview of a recommended homogenization and centrifugation procedure for preparing samples for analysis using TBS (Tris-buffered saline), TBST (TBS with addition of 1% Tritron-X100), and FA (formic acid) or GU-HCl (guanidine hydrochloride).

If ELISAs cannot be performed immediately after the *in vivo* experiment, it is advisable to delay tissue homogenization until the analysis can be conducted. This helps to minimize the release of proteases from the tissues. To further reduce protein degradation, protease inhibitors should be added to the homogenization buffer. It is recommended to perform homogenization of the samples at the same time to minimize variation between samples within the same experiments. After homogenization, samples should be stored at −80 °C, preferably in aliquots, if ELISAs are performed at different time points. To analyze Aβ from different locations (membrane-bound, non-membrane-bound, soluble, and insoluble), different buffers can be used sequentially. A schematic overview of the sample preparation process is shown in Figure 3 in the next section.

##### 2.1.1.2 Centrifugation speed

In addition to the choice of buffer, the speed of centrifugation also plays a crucial role in the separation profile. The homogenization process can impact insoluble aggregates and fibrils loosely associated with plaques, potentially causing them to partially solubilize. These aggregates require high centrifugation speeds to be fully pelleted. Most protocols use a centrifugation speed of 16,000 × *g*, which is achievable with most table top centrifuges. At this speed, some larger insoluble Aβ aggregates are pelleted, but many remain in the supernatant unless a higher speed is applied (Stern et al., 2023). Speeds of 100,000 × *g* or even 475,000 × *g* are needed to remove most of the larger aggregates, leaving primarily soluble aggregates or monomers in the supernatant (Stern et al., 2023). Aggregates detectable by Lecanemab, a monoclonal antibody specific for Aβ protofibrils and aggregated forms of Aβ (Johannesson et al., 2024), are only pelleted at speeds exceeding 100,000 × *g*. However, these aggregates can still be detected even after centrifugation at this speed. Lecanemab binds to both soluble and insoluble aggregates, but the efficiency of detection may vary depending on the size and solubility of the Aβ species in the sample. For example, larger aggregates may require higher centrifugation speeds for pelleting, whereas smaller soluble aggregates may remain in the supernatant.

Higher speeds (e.g., 100,000 × *g* or beyond) are necessary to separate larger aggregates, leaving behind predominantly soluble Aβ species in the supernatant. For a more comprehensive analysis, different fractions can be analyzed separately. Figure 3 provides a schematic overview of the homogenization and centrifugation process used to separate different pools of Aβ. This approach maximizes the information obtained from the sample, enabling researchers to study distinct forms of Aβ, such as monomers, oligomers, protofibrils, and plaques, providing a deeper understanding of the sample’s aggregation profile.

##### 2.1.1.3 Dilution

The quantity of aggregates in the analyzed sample is influenced by factors such as animal model, transgenic line, brain region, and disease progression. In genetically modified mice with Aβ pathology, aggregate levels generally increase with age. However, this trend may not apply to all types of aggregates, as some may be more abundant in younger mice. Prior to determining the appropriate dilution, it is useful to estimate the expected amounts of aggregates in the sample to select an appropriate dilution factor. After the homogenization and before analysis, it is crucial to dilute the homogenate accordingly. The ideal dilution should be chosen on a case-to-case basis. For assays with a broader detection range, such as those using Mesoscale Technology, the dilution factor becomes less critical. In general, rapid handling and maintaining samples at low temperatures will minimize the risk of both degradation and aggregation.

#### 2.1.2 General ELISA set up

**FIGURE 4:**
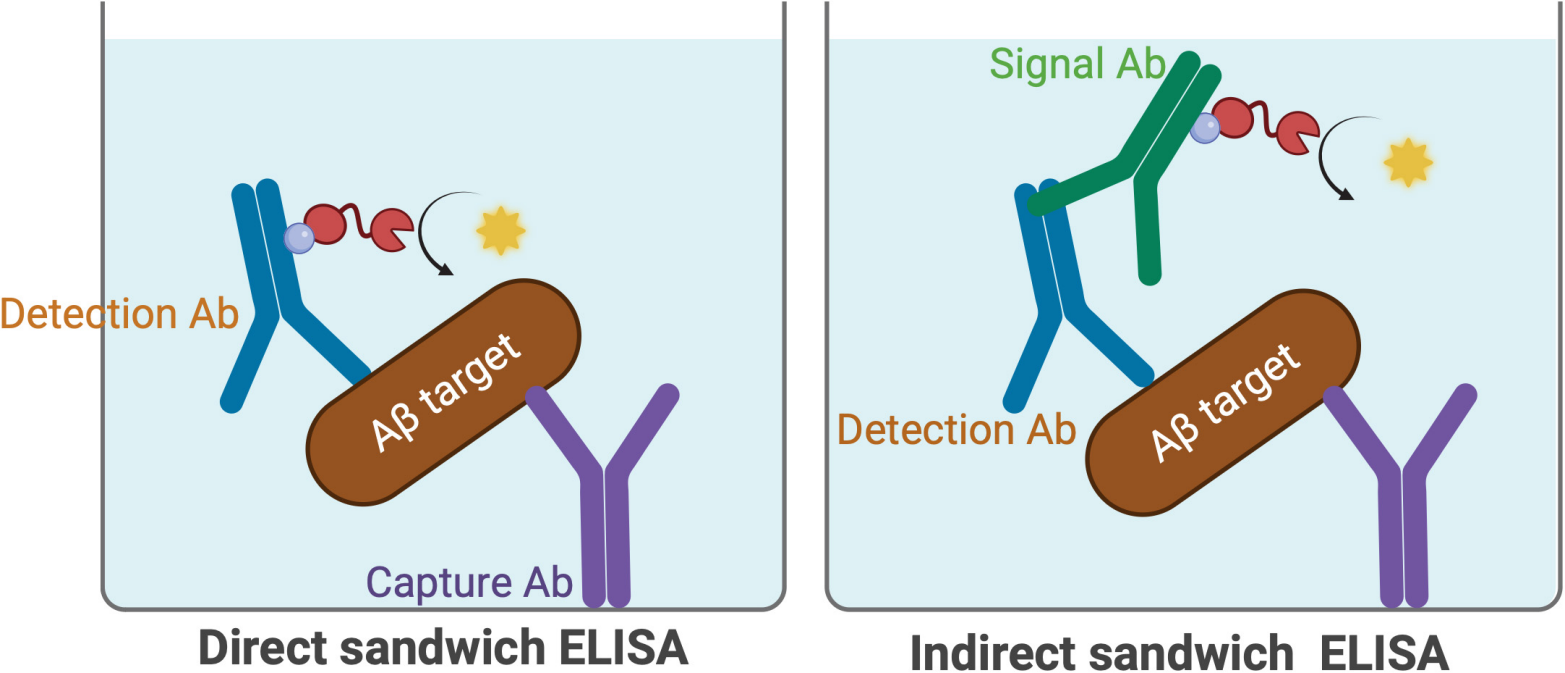
Schematic illustration of direct and indirect sandwich ELISA. In the direct sandwich ELISA the detection antibody is directly conjugated to an enzyme or a tag such as biotin, enabling the development of a detectable signal. In the indirect sandwich ELISA, an unlabeled detection antibody is used, followed by an enzyme-labeled secondary antibody that binds to the detection antibody, resulting in signal amplification.

An ELISA can be performed in several ways, including direct ELISA, sandwich ELISA, competitive ELISA, and inhibition ELISA. Of these, the sandwich ELISA is the most suitable for analysing protein levels in homogenate or serum and can be conducted in either a direct or indirect format (see Figure 4). In a direct sandwich ELISA, the detection antibody is directly labeled with detectable tag. In contrast, an indirect sandwich ELISA requires an enzyme-labeled secondary antibody (signal antibody) that binds to the detection antibody. This paper will focus on these two types of ELISA. It is crucial to ensure that the signal antibody does not cross-react with the capture antibody, so careful antibody selection is essential to minimize this risk, along with appropriate control experiments.

#### 2.1.3 General considerations about antibodies

##### 2.1.3.1 Sensitivity

Numerous methods have been developed to enhance the sensitivity of ELISA, enabling the detection of very low amounts of target proteins. Traditional ELISAs have served as the foundation for several advanced, automated, and ultrasensitive immunoassay technologies. Notable examples include the single-molecule array (SiMoA) by Quanterix (Rissin et al., 2010), Lumipulse by Fujirebio (Gili et al., 2021) ELISA-based Meso Scale Discovery (MSD) electrochemiluminescence assays by Meso Scale Diagnostics, LLC, and the Gyrolab platform by Gyros Protein Technologies. However, these approaches, while highly useful for detecting low signals and enabling standardized assays, are not discussed in detail within the scope of this paper.

**FIGURE 5:**
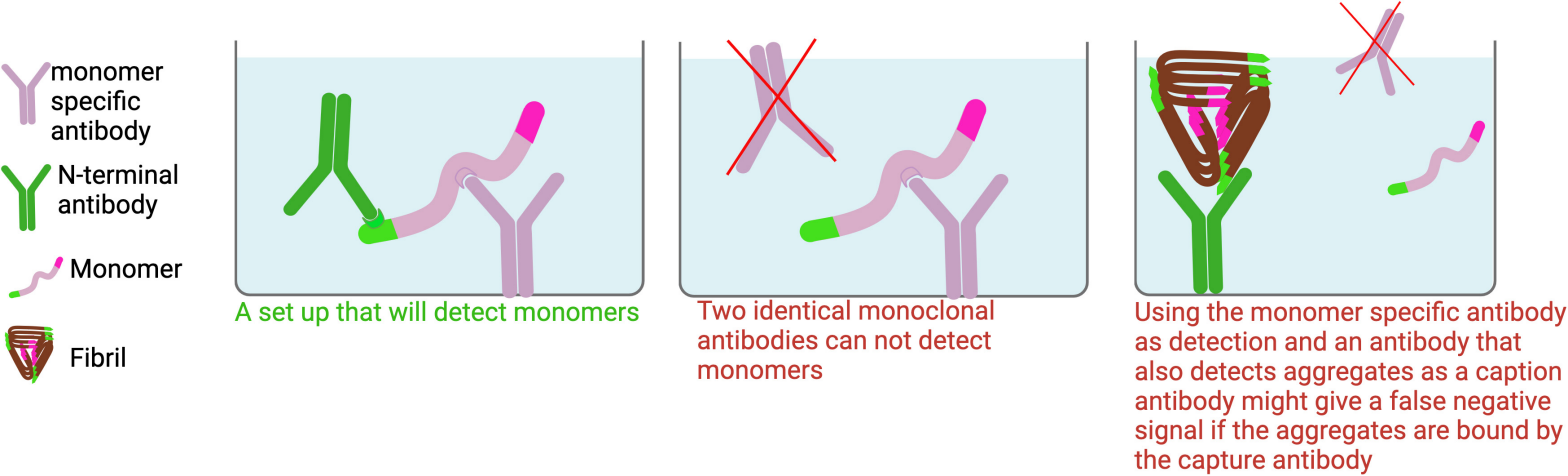
Monomer ELISA. To detect Aβ monomers in tissue homogenates, a monomer-specific antibody should be used as the capture antibody to minimize signal interference from aggregates. For optimal specificity, the capture and detection antibodies should target different epitopes on Aβ. While polyclonal antibodies may be suitable for detection, identifying a truly monomer-specific polyclonal antibody for capture is unlikely.

Simple strategies to slightly enhance the signal in an ELISA could be using a polyclonal antibody as the detection antibody. However, a polyclonal antibody can only bind to a few sites on an Aβ molecule due to its small size, resulting only in a slight signal amplification (Figure 5). However, if the polyclonal antibody binds to a small epitope of Aβ, amplification may not occur. None of the polyclonal antibodies listed in Table 1 are designed to bind large regions of Aβ, but they are effective in targeting specific small epitopes. A greater amplification is possible using a polyclonal antibody as a signal antibody in indirect sandwich ELISAs.

**TABLE 1:** List of well-characterized antibodies frequently used in our lab. While other antibodies targeting the same regions are available, those listed below have been carefully tested and characterized in our ELISA setups. If these alternative antibodies are specific to their targets, they would likely yield similar results in ELISAs.

Antibody abbreviation	Which types of Aβ does it bind to	Alternative name	Mono/ Poly-clonal	Is the antibody sequence available	Antigen	Binding region	Detects Aβ from these species	Produced by/sold by/reference
82E1- N terminal Aβ	Monomers and aggregates. Soluble and fibrillar Aβ. Aβ 1-X. Does not bind Aβ 2-X or APP		Monoclonal	Yes	Human Aβ (Bateman et al., 2023; Bateman et al., 2023; Bode et al., 2019; Fawzi et al., 2008; Gallego Villarejo et al., 2022; Grant et al., 2007; Huang and Liu, 2020; Kayed and Lasagna-Reeve, 2013; Larini and Shea, 2012; Lord et al., 2009; Nguyen et al., 2022; Ruttenberg and Nowick, 2024; Sengupta et al., 2016; Sevigny et al., 2016; Sims et al., 2023; Yang et al., 2023)	N-terminal (first amino acids)	Human, does not cross react with mouse or rat (Alzforum)	Is commercially available. (Horikoshi et al., 2004)
3D6- N terminal Aβ	Monomers and aggregates. Soluble and fibrillar Aβ. Aβ 1 -X. Does not bind Aβ 2-X or APP	Bapineuzumab is the humanized version	Monoclonal	Yes		N-terminal, AA1-5 of Aβ.	Human and mouse	Ours is in house produced, but is commercially available. (Johnson-Wood et al., 1997)
6E10 Close to N-terminal Aβ	Monomers and aggregates. Soluble and fibrillar Aβ Binds Aβ X-40/42. Binds APP		Monoclonal			AA5-10	Human (less well to murine)	Is commercially available. (Sloane et al., 1997)
Donanemab N-terminally truncated with pyroglutamate Aβ	Aβ(p3-X)	LY3002813	Monoclonal	Yes				Is commercially available. (Demattos et al., 2012)
4G8 Mid Aβ	Monomers and aggregates. Soluble and fibrillar Aβ. Binds N-terminally truncated. Binds APP		Monoclonal		Aβ juxta membrane EC domain AA 17-24	epitope lies between aa 18-22, but does not bind to for instance the arctic mutation	Dog, Human, Mouse/Rat others not tested	Is commercially available. (O’Connor et al., 2008)
M266 Monomeric Aβ	Monomers, soluble. Unable to bind fibrillar (Sumner et al., 2018)	Solanezumab is the humanized version	Monoclonal	Yes		Human AA 13-28 Or perhaps AA 16-24 LVFFAEDCG	Human. Likely also binds Mouse/Rat Do not know about other.	Ours is in house produced. Is commercially available. (DeMattos et al., 2001)
1A10- C terminal Aβ 40	Aβ X-40 specific, does not bind Aβ X-42		Monoclonal			AA35-40	Human, mouse, Rat	Is commercially available. (Horikoshi et al., 2004)
H31L21- C-terminal Aβ 42	Aβ X-42 specific, does not bind Aβ X-40, AβX-43		Monoclonal		AA707-713 = Antigen AA 36-42 VGGVVIA	Binds to C-terminal of Aβ.	Human and mouse	ThermoFisher Cat #700254
A11- Aβ and other oligomers with hairpin	Oligomers with hairpin. Also binds other types of oligomers with hairpin (like alpha synuclein). Does not bind monomers or mature fibrils.		Polyclonal	Not possible				Is commercially available. (Kayed et al., 2003)
mAb158- Aβ protofibrils	Detects protofibrils or larger. Binds weaker to insoluble aggregates. Binds with avidity, i.e., does not bind strongly to monomers and small oligomers.	Lecanemab is the humanised version	Monoclonal	Yes		Binds to AA 3-8		Ours is in house produced. (Englund et al., 2007)
Clone M3.2- Mouse and rat Aβ (close to N-terminal)	Is specific for murine/rat Aβ. Also detects murine/rat APP. Does not detect human Aβ.		Monoclonal			Binds to the 16 first AA (the only region that differs between mouse and human)	Rat and mouse. Does not bind to human	Nordic biosite, 805701
mAb27- Arctic Aβ	Binds specifically to Aβ with the Arctic mutation (APPE22G)		Monoclonal			Arctic Aβ		(Lord et al., 2009)

##### 2.1.3.2 Antibodies with selectivity based on the C-terminus of Aβ

In the ELISAs described in this paper, distinguishing between Aβ38, Aβ40 and Aβ42 is crucial. These proteins are highly similar, with Aβ42 differing from Aβ40 is also in the Aβ42 only by two additional amino acids at the C-terminus. As a result, developing an antibody specific to Aβ40 and Aβ38 is more challenging than one for Aβ42, as the latter provides more distinct binding sites. The same is of course true also for other C- and N-terminal truncations. When the difference between the antigens is minimal, well-characterized monoclonal antibodies are preferred. For polyclonal antibodies, a rigorous selection process is required.

The choice of sample, such as tissue homogenates, is just as important as the antibody used in the analysis. Detecting Aβ42 in a homogenate that predominantly contains Aβ40 is more challenging, as the higher concentration of Aβ40 can contribute to non-specific signals. In contrast, detecting Aβ42 is easier in samples where it is the dominant species. For example, in aged tg-ArcSwe brain, Aβ40 predominates, while in aged APP^NL–G–F^ Aβ42 may be present at levels up to 100 times higher than Aβ40, and there is also a lot of Aβ38. This is due to the Iberian mutation, which shifts the production toward Aβ42.

##### 2.1.3.3 Antibodies with selectivity between different types of aggregates

Considerable effort has been dedicated to developing antibodies that selectively recognize aggregated forms of proteins. Among the most well-known are Lecanemab and Aducanumab, both of which bind avidity-driven interactions, exhibiting stronger binding to aggregated Aβ species compared to monomers, which they cannot bind with avidity (Lannfelt et al., 2014; Sevigny et al., 2016). Although they retain some affinity for oligomeric forms, this binding is considerably weaker, indicating they are not truly aggregate-specific. Rofo et al., it is reported that Lecanemab binds with avidity to aggregates composed of 50-mers and larger, supporting the idea that its selectivity is primarily driven by multivalent interactions with large aggregates (Rofo et al., 2021).

##### 2.1.3.4 Selecting the right antibodies for accurate Aβ detection

When purchasing an antibody specific to a particular type of Aβ, it is important not to rely solely on the information provided by the supplier. Typically, the data given by the company refers to the antigen used to generate the antibody. For example, if Aβ40 monomer is used as the antigen, the resulting polyclonal antibodies will bind to Aβ40 as well as Aβ42 and other Aβ variants, since they all share similar regions. To ensure true specificity for Aβ40, the antibody’s purification and validation process must be thoroughly assessed. Were steps taken to remove antibodies that cross-react with Aβ42? Has it been confirmed that the antibody does not bind to Aβ42? For homogenate analysis, the antibody should have been tested in techniques like western blotting, using homogenates or cell lysates, and should show only a single band of the expected size. However, it is important to note that SDS-PAGE gels typically cannot differentiate Aβ40 from Aβ42. Often, antibodies will show multiple bands in western blot experiments, and using purified proteins for validation will not reveal how the antibody behaves with endogenous proteins in homogenates. Additionally, western blotting uses denatured proteins, so antibodies may interact differently with denatured versus non-denatured proteins. Therefore, selecting the right antibodies for Aβ ELISAs requires careful consideration and validation to ensure accurate and specific detection.

##### 2.1.3.5 Biotinylation of antibodies

One challenge when setting up ELISAs is if the capture and detection antibody originate from the same species. If they do, the secondary antibody used for signal detection may bind to both antibodies, leading to non-specific signals. When antibodies from different species are not available, a practical solution is to biotinylate the detection antibody.

Usually, antibodies are biotinylated by incubation with a 20-fold molar excess of 10 mM biotin solution (cat. no. 119616-38-5, Thermo Scientific) at room temperature for 30 min. To remove unbound biotin, buffer exchange to 1xPBS is performed by using Zeba spin desalting columns (7K, cat. no. 89883, Thermo Fisher). The protein concentration of the biotinylated antibody can be determined by using a NanoDrop spectrophotometer (Nanodrop 200C, Thermo Scientific). The quality and efficiency of biotinylation can be assessed by ELISA and Western blot, using avidin-HRP (cat. no. 18-4100-51, Thermo Scientific) as a detection reagent.

##### 2.1.3.6 Antibodies commonly utilized in our research

There is a wide range of antibodies available for Aβ detection in ELISAs, and it is impossible to compile an exhaustive list. However, after testing many options, we have identified several antibodies that work well in our ELISA setups. The antibodies we most frequently use for Aβ detection are listed in Table 1, along with a brief characterization of each. This list is by no means comprehensive but reflects the antibodies that have consistently performed well in our experiments. To improve clarity, we would like to explain the terminology used in this manuscript: We use “X” to indicate that the peptide length is not fixed at that terminus. For example, Aβ X-40 refers to peptides that end at amino acid position 40, but may start at any N-terminal position. Conversely, Aβ 1-X indicates peptides that start at position 1, with variable C-terminal lengths. When referring to Aβ40, we mean peptides that terminate at position 40, regardless of their N-terminal start site and is often used as a simplified notation.

## 3 Methods

### 3.1 Optimizing ELISA setups for selective detection of different Aβ Species

Designing an ELISA to selectively detect specific forms of Aβ peptides necessitates careful consideration of the structural characteristics and epitope accessibility of these aggregates. Monomeric Aβ peptides typically present a single accessible binding site for monoclonal antibodies, whereas dimers and larger aggregates offer multiple binding sites. When Aβ transitions from monomers to oligomers and fibrils, the conformation of the peptide changes, leading to the exposure of different epitopes. For instance, the N-terminal region (amino acids 1–19) of Aβ1–42 oligomers have been identified as an immunodominant region (Dalgediene et al., 2013). However, in aggregated forms, certain regions of Aβ may become conformationally altered or sterically hindered, potentially masking epitopes and impeding antibody binding.

When setting up an ELISA, it is crucial to define which forms of Aβ the assay is intended to detect. Monomers have only one accessible binding site for a monoclonal antibody, whereas dimers and larger aggregates present multiple binding sites, enabling different assay configurations. Below is a guide on how to configure ELISAs to selectively detect specific Aβ species, along with key considerations to ensure accurate and reproducible results.

#### 3.1.1 Monomers (all Aβ, AβX-40 and AβX-42)

To detect monomers in an ELISA, the same monoclonal antibody cannot be used for both capture and detection, as a monomer only has one accessible binding site (Figure 5). For detecting monomers of different isoforms, a monomer-selective capture antibody (e.g., m266, Table 1) should be paired with a C-terminal specific antibody (e.g., H31L21 or 1A10) targeting the desired Aβ isoform. This combination ensures specific detection of monomers while avoiding cross-reactivity with aggregates. If the sample contains both aggregates and monomers, using a non-selective capture antibody that binds to both forms may result in preferential binding to aggregates, which could block monomer detection and lead to underestimation of monomer levels.

#### 3.1.2 Aβ dimers and larger aggregates, excluding monomers

**FIGURE 6:**
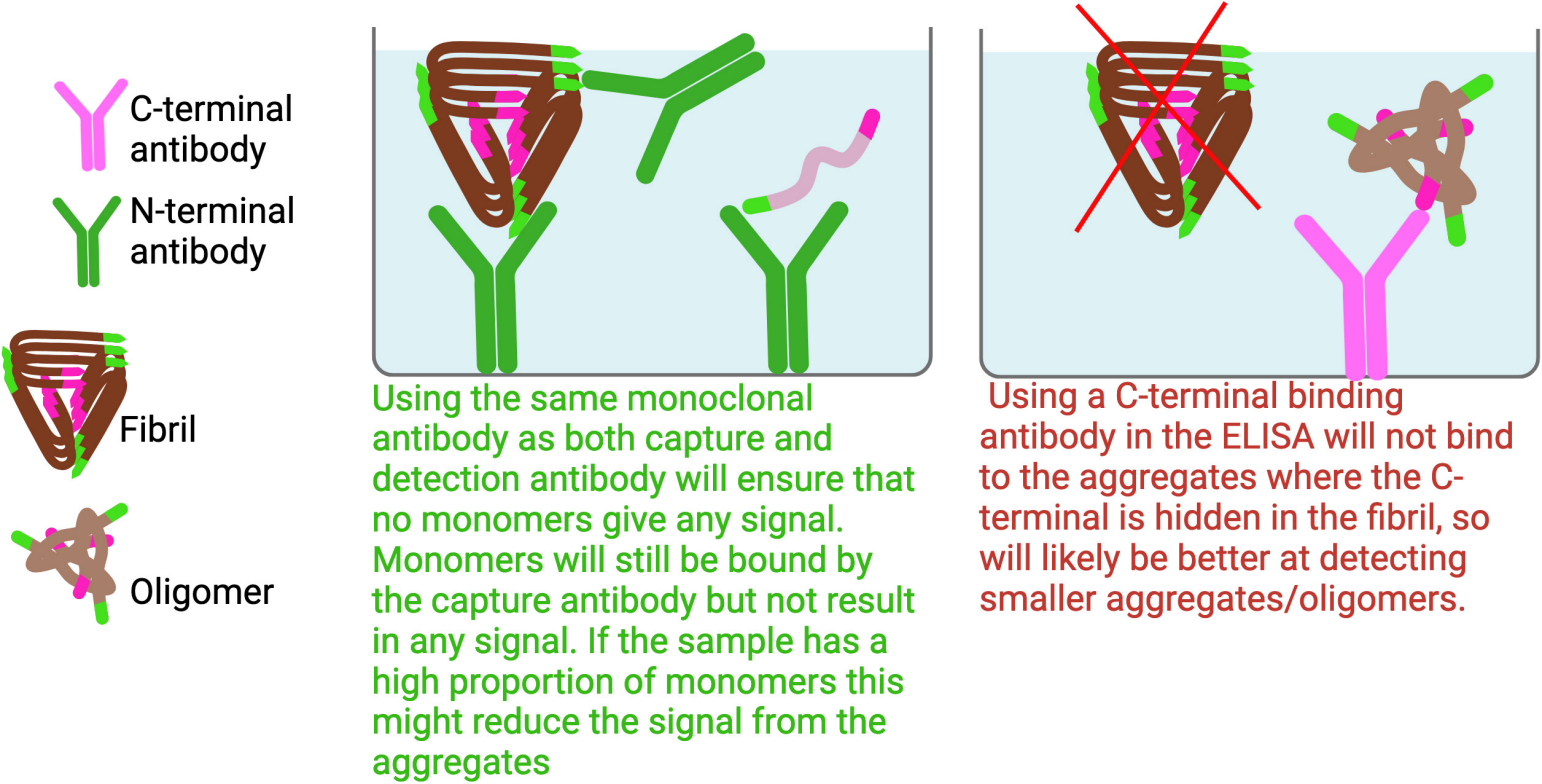
Dimer and larger Aβ aggregates ELISA. To effectively capture a broad range of Aβ aggregates, it is advisable to avoid using antibodies that target the C-terminal or mid regions of Aβ, as these epitopes are often buried within the aggregated structures and may not be accessible for binding.

To selectively detect Aβ aggregates starting from dimers while excluding monomers, the same monoclonal antibody should be used for both capture and detection. Monomers cannot bind the same monoclonal antibody twice, allowing for selective detection of aggregates (Figure 6). Because the N-terminus is typically accessible in most Aβ aggregates, an antibody targeting the N-terminal region is recommended for this setup to maximize aggregate detection across a broad range of species.

#### 3.1.3 Detection of different Aβ isoforms in aggregates with ELISAs

Detecting specific Aβ isoforms within aggregates using ELISAs presents several challenges. It is often difficult to determine whether the detected aggregates are primarily composed of Aβ40, Aβ42, or a mixture of both. Two key factors contribute to this difficulty: First, the C-terminal region of Aβ is often hidden within aggregates (Roher et al., 2000; Stenh et al., 2005). Second, aggregates often contain of a heterogenous mix of Aβ40, Aβ42, and other Aβ isoforms. Even when using an antibody specific to Aβ42 as a capture antibody, it may still bind aggregates that are primarily composed of Aβ40, due to the close proximity of epitopes in the aggregated form.

Using C-terminal-specific antibodies for both capture and detection could introduce bias, as only certain aggregates may be efficiently detected, potentially leading to misleading conclusions. A possible workaround is to first separate the aggregated Aβ species, for example using size-exclusion chromatography or immunoprecipitation with an aggregate-specific antibody, and then dissociate the aggregates into monomers using a denaturing method such as formic acid treatment. This allows subsequent analysis of the isoform composition at the monomeric level.

#### 3.1.4 Challenges in detection of oligomeric Aβ in ELISA

Setting up an ELISA to specifically detect oligomeric Aβ is challenging due to the structural heterogeneity and transient nature of these species. Oligomers are typically unstable and short-lived, making it difficult to generate antibodies that selectively recognize unique epitopes. One approach to overcome this challenge is the use of stabilized oligomers. For example, a disulfide-linked trimer has been engineered, and an antibody has been developed that specifically binds to this stabilized form (Sandberg et al., 2010). Another well-characterized antibody, A11, binds to a beta-hairpin motif commonly found in early oligomers (Kayed et al., 2003). However, this structural motif is not unique to Aβ and is also present in aggregates of other proteins, such as alpha-synuclein, making the A11 antibody cross-reactive.

**FIGURE 7:**
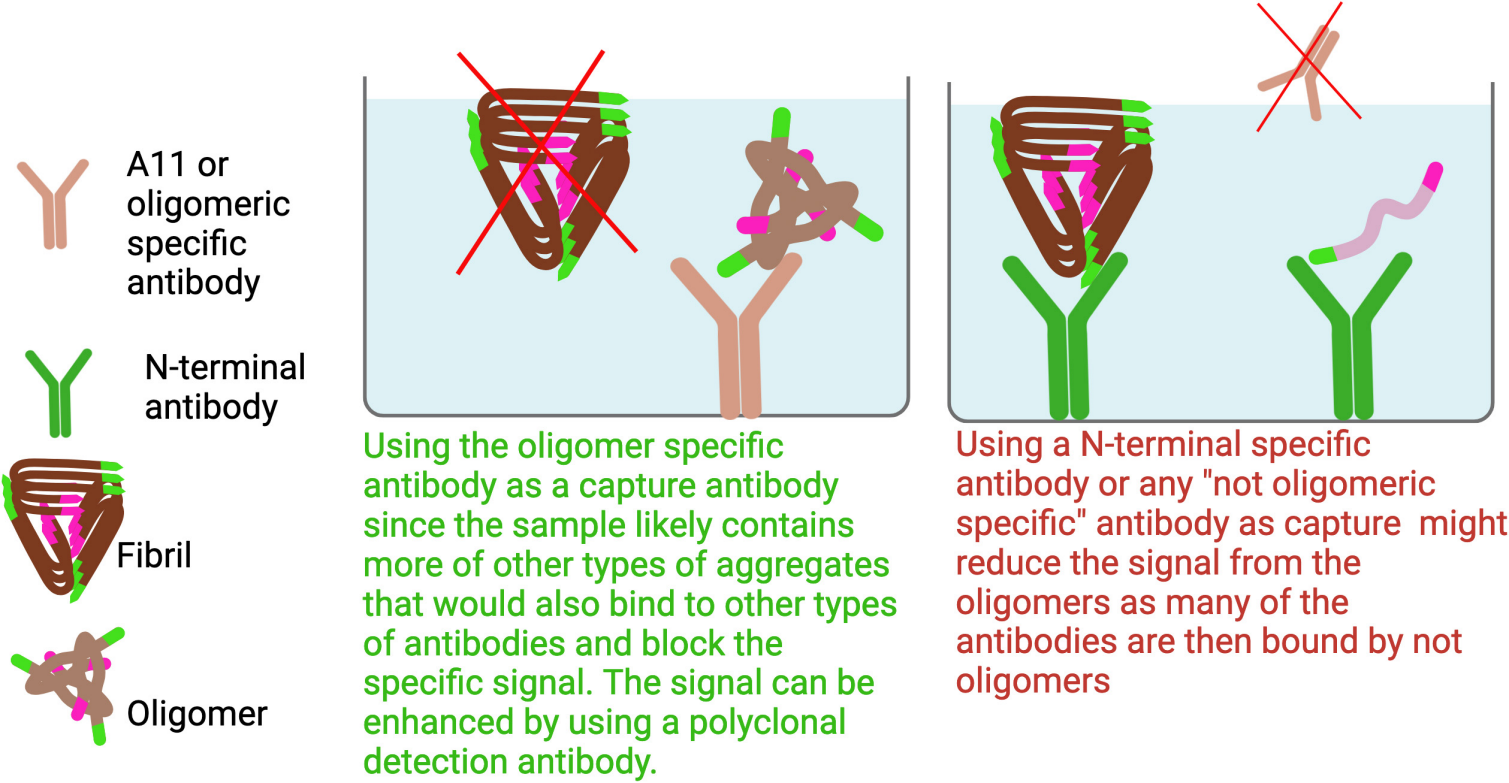
To specifically detect oligomers, an oligomer-selective antibody should be used as the capture antibody to avoid binding larger aggregates that could dilute the signal. Since the C-terminal region is generally more exposed in smaller aggregates and oligomers, using a C-terminal antibody for both capture and detection can enhance selectivity for these forms. Additionally, high-speed centrifugation can enrich for soluble oligomers by pelleting larger aggregates.

In ELISAs aimed at detecting oligomers, it is essential to use an oligomer-specific antibody as the capture antibody. This is because oligomers are typically present in lower quantities compared to larger aggregates, and using other antibodies as the capture antibody could result in epitope masking or signal suppression due to preferential binding of more abundant species (Figure 7).

Another potential setup involves using the same monomeric antibody for both capture and detection, targeting epitopes at the C-terminus of Aβ aggregates. Since the C-terminus is often buried within larger aggregates, this configuration would predominantly detect smaller aggregates (Roher et al., 2000; Stenh et al., 2005). Moreover, when samples are ultracentrifuged at speeds greater than 100,000 x *g*, larger aggregates are pelleted, enriching the supernatant for smaller, soluble aggregates. As a result, an ELISA designed to detect aggregates in such supernatants would primarily capture these smaller, soluble aggregates.

#### 3.1.5 Detection of Aβ with the N- and C-terminal available

To detect all forms of Aβ with accessible N- and C-termini, using a C-terminal antibody as the capture antibody is recommended. This avoids a scenario where the capture antibody is predominantly bound by the N-terminus of Aβ in aggregates, which could sterically hinder detection and thus reduce signal strength. Aβ oligomers often differ from monomers structurally, with the C-terminal region buried in a hydrophobic core, reducing accessibility. As larger aggregates tend to hide the C-terminus even further, this strategy improves detection sensitivity across aggregation states of Aβ (Roher et al., 2000; Stenh et al., 2005).

#### 3.1.6 Detection of N-terminal truncations in Aβ aggregates

The significance of N-terminal truncations has been underscored by the success of the antibody Donanemab in recent clinical trials. This antibody targets N-terminally truncated pyroglutamate-modified Aβ (pE3Aβ) (Sims et al., 2023). N-terminal truncations are commonly found in plaques of Alzheimer’s patients, although their presence in smaller aggregates and oligomers remains less understood. To selectively capture these truncations, Donanemab should be used as the capture antibody. Additionally, Donanemab can also serve as the detection antibody if the aggregates contain multiple pE3Aβ molecules. Otherwise, an antibody binding close to the N-terminal, but excluding AA1-2, should be chosen.

It is important to note that N-terminally truncated Aβ will not be detected by antibodies such as 3D6 and 82E1, which bind to the very N-terminal of Aβ. As a result, these truncated forms will not contribute to the results in many of the ELISAs described above and below.

#### 3.1.7 Human versus murine Aβ

Aβ analysis is commonly performed in genetically modified mice, which often feature altered APP expression. While wild-type mice can be used, transgenic models, such as those overexpressing APP, are more frequently employed. These models typically have one or more copies of the APP gene inserted into the genome. Since murine Aβ is less prone to aggregation, and many of these models are used with the aim to develop therapeutics for human diseases, human APP is used instead.

Two transgenic models that we commonly use, tg-ArcSwe and tg-Swe, express human Aβ under the brain-specific Thy1 promoter (Yokoyama et al., 2022). As a result, these models contain both murine and human Aβ in the brain, although human Aβ predominates, while only murine Aβ is found in the rest of the body. In these models, if the overexpression of APP is restricted to the brain, murine Aβ typically dominates in the blood, while human Aβ is more prevalent in the brain.

More recently, knock-in mouse models, such as App^NL–F^ and App^NL–G–F^ (Saito et al., 2014), have been developed. These mice, when bred homozygous, express only human Aβ, providing a more direct model for studying human-specific Aβ pathology.

#### 3.1.8 Fibrils made of Arctic mutation

Several genetic variants of Alzheimer’s disease (AD) involve mutations within the Aβ peptide. One such mutation is the Arctic mutation, which accelerates the rate of Aβ aggregation. Others are the Dutch and the Iowa mutation. Other mutations, like the Swedish mutation, occur outside the Aβ peptide. Arctic Aβ has been shown to fold differently in various brains but has in tg-ArcSwe mice been shown to fold the same way as in sporadic Alzheimer’s disease (Yang et al., 2023; Zielinski et al., 2023). The different folding suggests that it might be detected using different combinations of antibodies compared to other Aβ variants.

To specifically capture aggregates carrying the Arctic or other mutations in heterozygous mice, a mutation-specific antibody should be used as the capture antibody. In homozygous mice, where all Aβ aggregates contain the mutation, this approach ensures that only aggregates composed of the mutant form are captured, thereby increasing specificity and reducing background from wild-type Aβ.

Step by step ELISA protocol

1. Coat plates with 1 μg/mL of the capture antibody, either overnight at 4°C or 1 h at room temperature (RT).
2. Block wells by incubating for 2 h at RT with 1% (w/v) BSA in PBS
3. Wash wells 3 x times with ELISA washing buffer (1 x PBS with 0.05% Tween-20).
4. Prepare suitable standard curves in ELISA incubation buffer (EIB, 0.05% Tween-20, 1% BSA in PBS), keep on ice und use within 20 min.
5. Apply samples in duplicates to the ELISA plate and incubate at RT for 2 h (longer or shorter incubation times may be used dependent on optimization).
6. Wash wells 3 x times with ELISA washing buffer.
7. Dilute the detection antibody in EIB to a suitable concentration based on binding affinity. Incubate for 2 h at RT.
8. Wash wells 3 x times with ELISA washing buffer.
9. Add signal antibody diluted in EIB (e.g., streptavidin-HRP or IgG HRP-conjugated antibody, depending on detection setup).
10. Wash wells 3 x times with ELISA washing buffer.
11. Develop signal with K-blue^®^ aqueous TMB substrate and stop the reaction with 1 M H_2_SO_4_ in a 1:1 (v/v) ratio.
12. Measure absorbance at 450 nm using a multimode microplate reader.

### 3.2 Standard curves

Generating an ideal standard curve for an ELISA is challenging, and in many cases, it is not possible to create a standard curve that accurately reflects the concentration of a specific Aβ aggregate type. A tissue homogenate typically contains a mixture of various forms of Aβ, including Aβ1-38, Aβ1-40, Aβ1-42, Aβ1-43, N-terminally truncated Aβ (of the aforementioned isoforms), as well as both human and murine Aβ. Additionally, the sample will contain Aβ protofibrils, Aβ fibrils, monomeric Aβ, oligomeric Aβ, and larger aggregated Aβ.

Since standard curves usually represent only one specific isoform, they might consist of varying proportions of monomers and different aggregate types, which can complicate the interpretation of results.

Possible standard materials include monomeric Aβ1-40, monomeric Aβ1-42, aggregated Aβ, sonicated fibrils, or engineered Aβ forms that can generate stable oligomers. Aβ oligomers can also be used as a standard, but generating reproducible Aβ oligomers for use in standard curves is challenging. However, the selection of the most appropriate standard depends on the specific Aβ form being analyzed.

A freshly prepared standard curve of human Aβ1-40 monomers is less prone to aggregation and will predominantly consist of monomers. However, Aβ1-40 will begin to aggregate over time, so it is essential to prepare it fresh or check for aggregates before use.

In contrast, Aβ1-42, aggregates rapidly, so even a freshly prepared standard curve will likely contain some degree of aggregates. One option is to purify aggregated Aβ42 via size exclusion chromatography (SEC) to remove the monomeric fraction, though aggregation may resume afterward. Keeping peptide concentrations as low as possible will also help minimize aggregation.

When preparing standard curves for Aβ variants with mutations, such as the Arctic mutation, which aggregate even faster, it is crucial to ensure aggregation has not already occured prior to analysis. Achieving a standard curve without any aggregates might be impossible in such cases.

Aβ fibrils are generally too large to be used effectively in standard curves due to sedimentation. However, sonicated fibrils (PF-42) may be used as a more stable alternative.

#### 3.2.1 When detecting monomeric Aβ

In an ELISA where a monomer-specific capture antibody is used, only monomers will bind, and the corresponding monomeric standard curve (such as for Aβ1-40 or Aβ1-42 monomers) can be applied depending on the detection antibody. However, when using capture antibodies that bind both monomers and aggregates, or antibodies with different affinities for monomers and aggregates, the monomeric standard curve should be used with caution.

It is important to remember that antibodies can bind with *avidity*—stronger binding when the antibody binds with both its arms of the target. This phenomenon does not apply to monomers, as they can only bind one antibody molecule, but it is seen in aggregates, where antibodies can bind multiple sites on the same target, resulting in stronger binding (an example of this is Lecanemab).

#### 3.2.2 When detecting aggregates excluding monomers

When using the same monoclonal antibody for both capture and detection, the ELISA will only detect aggregates. However, in most cases, these antibodies will also bind monomers. If the homogenate being analyzed contains high amounts of monomers, a significant portion of the capture antibody will bind to the monomers, which will reduce the potential signal from the aggregates. This can result in an underestimated signal.

To avoid this reduction in signal, a standard curve that excludes monomers should be used. Using a monomer-free standard curve ensures that the measurement is not affected by the presence of monomers in the sample. However, if the homogenates contain varying amounts of monomers, this can introduce variability, potentially impacting the accuracy of comparisons between different samples.

#### 3.2.3 Preparation of a pre-aggregated Aβ42 and Aβ42 protofibrils

**Pre-aggregated Aβ 42:**

1. Incubation of 100 nM Aβ42 peptide (e.g., cat. no. SP-BA42-1, Innovagen) was performed at + 37°C approximately 3 h prior to ELISA analysis. Immediately before preparation of the standard curve, the sample was briefly centrifuged at 16,000 × *g* to remove insoluble aggregates.

**Aβ 42 protofibrils:**

1. Aβ42 peptide (e.g., cat. no. SP-BA42-1, Innovagen) was incubated at + 37°C for 3 h.
2. Following incubation, the sample was centrifuged at 16,000 × *g* for 10 min to remove insoluble aggregates.
3. The resulting supernatant was carefully collected, and Tween-20 was added to a final concentration of 0.6% (v/v).
4. The supernatant was then subjected to a size-exclusion chromatography using a Superdex 200 increase 10/300 GL column (cat. no. GE28-0009-44, Cytiva), pre-equilibrated with 0.6% (v/v) Tween-20 in 1xPBS (cat. no. 14190250, Thermo Fisher).
5. Elution was performed at a flow rate of 0.5 mL/min. Fractions were collected and analyzed by SDS-PAGE (4 – 12% Bis-Tris protein gel, cat. no. NW04125BOX, Invitrogen).
6. The molecular size of the eluted peaks was assessed by comparison to a calibration standard consisting of Thyroglobulin 669 kDa, Ferritin 440 kDa, Aldolase 158 kDa, Conalbumin 75 kDa, Ovalbulmin 43 kDa, Carbonic anhydrase 29 kDa, previously run on the column.
7. The concentration of Aβ42 protofibrils in the eluted fractions was determined using specific ELISAs (EAβdopf1-X – 3D6 - 3D6biot, EAβpf1-X – RmAb158-3D6biot).

### 3.3 Examples of ELISAs

In Table 2, which outlines the ELISA setups we frequently use, a suggested standard curve is also provided for each setup.

**TABLE 2:** Well-characterized ELISA setups for detecting distinct Aβ species and aggregates.

ELISA name/abbreviation *E* = ELISA *m* = monomer *d* = dimer *o* = oligomer *p* = protofibril *f* = fibril	Detection	Excludes	Not known	Antibodies used (capture-detection)	Suitable standard curve/positive control	Can total amount of the tested Aβ variant be determined in the sample?	Important to consider
EAβm1-X	Aβ monomers 1-X	aggregates, N-terminally truncated		m266 – 3D6	Aβ1-X (X > 24 AA)	Yes	
EAβmX-40	Aβ40 monomer (human, mouse) X-40	aggregates, Aβ42	arctic and other mutants	m266 – 1A10	AβX-40 monomer	Yes	In the formic acid (FA) fraction, which predominantly contains Aβ monomers, it is advisable to use the 1A10 antibody as the capture antibody to not dilute the signal caused by Aβ42 binding to m266.
EAβmX-42	Aβ42 monomer (human, mouse) X-42	aggregates, Aβ40		m266 – H31L21	AβX-42 monomer	Yes	In the FA fraction, which mainly contains Aβ monomers, the H31L21 antibody should be used as the capture antibody to avoid signal dilution by Aβ40 binding to m266. Aβ42 monomers are prone to aggregation.
EAβdopf1-X	Aβ 1-X dimers and larger aggregates	monomers, N-terminally truncated		3D6 – 3D6 82E1 – 3D6 82E1 – 82E1	PF-42 (in the standard curve, none of the capture antibodies are blocked by monomers)	maybe	A sample with high concentration of Aβ monomers some capture antibody may become occupied by these species.
EAβdopf1-X	Aβ X-X dimers and larger aggregates	monomers		4G8 – 4G8	As above	maybe	As above
EAβdopf (p3)-X	Aβ dimers and larger aggregates, with N-terminal truncations and pyroglutamate	Monomers, Not truncated Aβ		Donanemab – Donanemab			
EAβmdopf1-40	Aβ40 monomers, aggregates (mouse)	N-terminally truncated, Aβ42		82E1 – 1A10 3D6 – 1A10	Aβ1-40 monomer	no	Capture antibodies may also bind Aβ42 and other species not detected by the 1A10 antibody, reducing the signal relative to the standard curve equivalent to Aβ40 concentrations. This can lead to underestimation of total Aβ levels in mixed samples.
EAβmdopf1-42	Aβ42 monomers and aggregates with N- and C-terminal free	N-terminally truncated, Aβ40		82E1 – H31L21 3D6 – H31L21	Aβ1-42	no	Capture antibodies will also bind Aβ40 and other species not detected by H31L21, resulting in a lower signal than expected from the standard curve at equal Aβ42 concentration.
EAβpf1-X	Aβ aggregates, protofibrils and larger	Aβ monomers, small oligomers, N-terminally truncated		RmAb158 – 3D6 RmAb158 – 82E1	PF-42	no	If the homogenate contains high levels of Aβ monomers, they can saturate the capture antibodies, potentially leading to underestimation of Aβ levels compared to the standard curve.
EAβo1-X	oligomers with a beta hair pin	monomers and larger aggregates which have gone through the switch from beta hairpin to beta sheets, N-terminally truncated		A11 – 3D6 A11 – 82E1	Aβ oligomers	no	Generating Aβ oligomers for in use in standard curves is challenging, and the A11 antibody, which detects generic hairpin structures, may also bind to non- Aβ peptides. This can compete with Aβ binding at the capture antibody, reducing the true signal.
EAβmdopfArc1-X	Arctic Aβ40 and 42 monomers and aggregates	Aβ without Arctic mutation, N-terminally truncated		mAb27 – 82E1 mAb27 – 3D6	AβArc40	no	

## 4 Results

**FIGURE 8:**
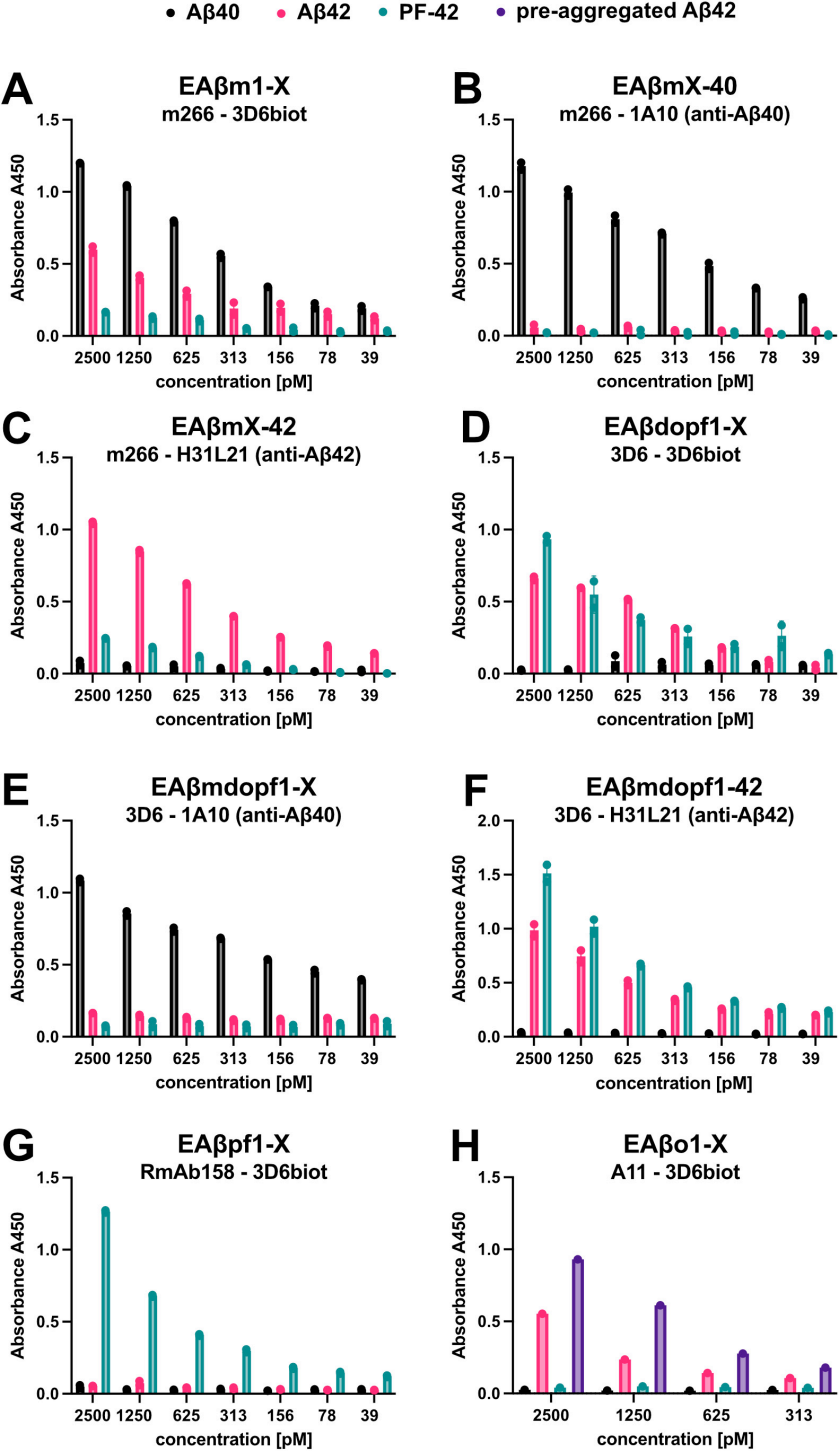
Well-characterized ELISA setups for detecting distinct Aβ species and aggregates. **(A–H)** Selected ELISA setups from Table 2 were evaluated for their ability to detect various Aβ species. Standard curves were generated using synthetic preparations of Aβ40 monomers, Aβ42 monomers, Aβ42 protofibrils (PF-42), and pre-aggregated Aβ42 (only in Figure 8H), but the results indicates that at the Aβ42 monomer standard curve does not stay monomeric. The abbreviations of the ELISA setups and the corresponding capture and detection antibodies are indicated in each graph. Data represent the mean with standard deviation of duplicates.

Selected ELISA setups from Table 2 were evaluated for their selectivity in detecting specific Aβ forms, using data generated by standard curves made from Aβ1-40 monomers, Aβ1-42 monomers, Aβ42 protofibrils (PF-42), and pre-aggregated Aβ42 as described in “see Section 3.2 Standard curves” (Figure 8).

It is evident in for instance the ELISA setup EAβdopf1-X (Figure 8D), where the monoclonal 3D6 antibody is used as both the capture and detection antibody, that the standard curve generated using Aβ42 monomers also contains an aggregated fraction. The Aβ42 standard curve exhibits a clear signal, suggesting that at least dimers are formed during the incubation process. This interpretation is further supported by the data presented in Figure 8A, which shows results from the ELISA setup EAβm1-X. In this assay, monomer-specific detection is achieved using the m266 antibody for coating and the 3D6 antibody for detection. Only monomers of Aβ40 and Aβ42 are detected under these conditions.

Notably, there is a difference in signal intensity between Aβ40 and Aβ42, indicating that Aβ42 is more prone to aggregation, which reduces its detectable monomeric form (Figure 8A). The aggregates formed by Aβ42 appear to be different from PF-42, as they are not detected in the ELISA setup EAβpf1-X, where RmAb158 is used for coating and 3D6 for detection (Figure 8G). This particular assay is designed to selectively detect Aβ fibrils, protofibrils, and larger assemblies, while excluding Aβ monomers, small Aβ oligomers, and N-terminally truncated Aβ variants.

In contrast, Figure 8H shows the results from the EAβo1-X ELISA, in which the conformation-specific A11 antibody is used for coating and 3D6 for detection. Here, pre-aggregated Aβ42 was included as a standard, and a detectable signal was observed, albeit weaker than that for the PF-42 standard. This suggests that Aβ42 monomers undergo aggregation during the ELISA incubation process, but the extent of aggregation may be limited. The weaker signal implies that longer incubation times may be necessary to allow the formation of larger aggregates or protofibrils comparable to PF-42. For further reference, in our recent publication (Metzendorf et al., 2025), we used the same homogenization procedure and a similar set of ELISA setups to characterize which Abeta variants were affected by the treatment.

## 5 Discussion

Enzyme-Linked Immunosorbent Assay (ELISA) is often perceived as a straightforward and accessible technique; however, achieving accurate and reliable results require careful attention to detail throughout the experimental setup. The success of an ELISA depends not only on the quality of the antibodies used but also on the choice of capture and detection reagents, sample preparation, and the specific characteristics of the analyte being studied. Some assays may be affected by incubation times and temperature, while others may not be sensitive to these conditions. Maintaining strict control over incubation parameters (including temperature) is therefore critical, as even small variations can impact assay performance.

Commercial Aβ ELISA kits are widely used but often lack transparency regarding antibody specificity, epitope recognition, and sensitivity to different Aβ species. This can lead to misinterpretation, particularly when attempting to distinguish between monomeric, oligomeric, and fibrillar forms. Many kits recommend solubilizing samples directly in strong denaturants such as guanidine hydrochloride (GuHCl), which prevents the separation of soluble, membrane-associated, and insoluble Aβ fractions, thereby masking biological relevant distinctions.

Kits that claim to detect specific Aβ lengths often rely on C-terminal antibodies, which may fail to bind larger aggregates in samples prepared with TBS and TBST, leading to preferential detection of smaller species. In addition, commonly used N-terminal capture antibodies often favor the most abundant Aβ form, increasing the risk of underestimating less prevalent or structurally hidden species. These limitations highlight the importance of critically evaluating antibody configurations and sample preparation methods when interpreting Aβ ELISA results, particularly in translational or mechanistic studies.

Differences in Aβ sequence or structural conformation across species may also influence antibody binding and detection efficiency. As a result, quantification of Aβ species in non-human models may not always directly reflect their abundance or forms in human samples. These potential differences should be taken into account when interpreting cross-species data, especially in studies aiming to translate findings from experimental models to human disease contexts.

By tailoring ELISA setups to differentiate between various forms of Aβ, such as monomers, oligomers, aggregates, and different Aβ forms, more detailed and meaningful data can be extracted from the samples. The strategies and protocols outlined above offer enhanced sensitivity and specificity compared to standard ELISA methodologies, providing a more comprehensive approach to studying Aβ and its role in Alzheimer’s disease and other neurodegenerative conditions. These improved setups ensure that data can be interpreted with greater confidence, advancing our understanding of protein aggregation and its pathological consequences.

In addition to their utility in mechanistic studies, these protocols have potential applications in therapeutic development. Specifically, they can be integrated into drug screening pipelines to evaluate the selectivity and efficacy of candidate therapeutics targeting specific Aβ species. Since the method allows for detection of key aggregation intermediates such as oligomers and protofibrils, it can be used to monitor drug-induced shifts in Aβ aggregation states. Furthermore, its compatibility with soluble and insoluble fractions makes it suitable for use in *in vitro* and *in vivo* models. These features support the method’s applicability in preclinical drug screening workflows aimed at identifying compounds that modulate Aβ pathology in species-specific and aggregation state-specific manner.

## Data Availability

The raw data supporting the conclusions of this article will be made available by the authors, without undue reservation.

## References

[B1] Bateman R. Smith J. Donohue M. Delmar P. Abbas R. Salloway S. (2023). Two phase 3 trials of Gantenerumab in early Alzheimer’s disease. *N. Engl. J. Med.* 389 1862–1876. 10.1056/NEJMoa2304430 37966285 PMC10794000

[B2] Bode D. Freeley M. Nield J. Palma M. Viles J. (2019). Amyloid-β oligomers have a profound detergent-like effect on lipid membrane bilayers, imaged by atomic force and electron microscopy. *J. Biol. Chem.* 294 7566–7572. 10.1074/jbc.AC118.007195 30948512 PMC6514634

[B3] Celej M. Sarroukh R. Goormaghtigh E. Fidelio G. Ruysschaert J. Raussens V. (2012). Toxic prefibrillar α-synuclein amyloid oligomers adopt a distinctive antiparallel β-sheet structure. *Biochem. J.* 443 719–726. 10.1042/BJ20111924 22316405

[B4] Dalgediene I. Lasickiene R. Budvytyte R. Valincius G. Morkuniene R. Borutaite V. (2013). Immunogenic properties of amyloid beta oligomers. *J. Biomed. Sci.* 20:10. 10.1186/1423-0127-20-10 23432787 PMC3599114

[B5] DeMattos R. Bales K. Cummins D. Dodart J. Paul S. Holtzman D. (2001). Peripheral anti-A beta antibody alters CNS and plasma A beta clearance and decreases brain A beta burden in a mouse model of Alzheimer’s disease. *Proc. Natl. Acad. Sci. U S A.* 98 8850–8855. 10.1073/pnas.151261398 11438712 PMC37524

[B6] Demattos R. Lu J. Tang Y. Racke M. Delong C. Tzaferis J. (2012). A plaque-specific antibody clears existing β-amyloid plaques in Alzheimer’s disease mice. *Neuron* 76 908–920. 10.1016/j.neuron.2012.10.029 23217740

[B7] Englund H. Sehlin D. Johansson A. Nilsson L. Gellerfors P. Paulie S. (2007). Sensitive ELISA detection of amyloid-beta protofibrils in biological samples. *J. Neurochem.* 103 334–345. 10.1111/j.1471-4159.2007.04759.x 17623042

[B8] Fawzi N. Kohlstedt K. Okabe Y. Head-Gordon T. (2008). Protofibril assemblies of the arctic, Dutch, and flemish mutants of the Alzheimer’s Abeta1-40 peptide. *Biophys. J.* 94 2007–2016. 10.1529/biophysj.107.121467 18032553 PMC2257882

[B9] Gallego Villarejo L. Bachmann L. Marks D. Brachthäuser M. Geidies A. Müller T. (2022). Role of intracellular amyloid β as pathway modulator, biomarker, and therapy target. *Int. J. Mol. Sci.* 23:4656. 10.3390/ijms23094656 35563046 PMC9103247

[B10] Gao Y. Wennmalm S. Winblad B. Schedin-Weiss S. Tjernberg L. (2021). Live Cell FRET imaging reveals Amyloid β-peptide oligomerization in hippocampal neurons. *Int. J. Mol. Sci.* 22:4530. 10.3390/ijms22094530 33926107 PMC8123703

[B11] Gili A. Paggi R. Russo C. Cenci E. Pietrella D. Graziani A. (2021). Evaluation of Lumipulse^®^ G SARS-CoV-2 antigen assay automated test for detecting SARS-CoV-2 nucleocapsid protein (NP) in nasopharyngeal swabs for community and population screening. *Int. J. Infect. Dis.* 105 391–396. 10.1016/j.ijid.2021.02.098 33647511 PMC7908845

[B12] Grant M. Lazo N. Lomakin A. Condron M. Arai H. Yamin G. (2007). Familial Alzheimer’s disease mutations alter the stability of the amyloid beta-protein monomer folding nucleus. *Proc. Natl. Acad. Sci. U S A.* 104 16522–16527. 10.1073/pnas.0705197104 17940047 PMC2034231

[B13] Horikoshi Y. Sakaguchi G. Becker A. Gray A. Duff K. Aisen P. (2004). Development of Abeta terminal end-specific antibodies and sensitive ELISA for Abeta variant. *Biochem. Biophys. Res. Commun.* 319 733–737. 10.1016/j.bbrc.2004.05.051 15184044

[B14] Huang Y. Liu R. (2020). The toxicity and polymorphism of β-Amyloid oligomers. *Int. J. Mol. Sci.* 21:4477. 10.3390/ijms21124477 32599696 PMC7352971

[B15] Johannesson M. Söderberg L. Zachrisson O. Fritz N. Kylefjord H. Gkanatsiou E. (2024). Lecanemab demonstrates highly selective binding to Aβ protofibrils isolated from Alzheimer’s disease brains. *Mol. Cell. Neurosci.* 130:103949. 10.1016/j.mcn.2024.103949 38906341

[B16] Johnson-Wood K. Lee M. Motter R. Hu K. Gordon G. Barbour R. (1997). Amyloid precursor protein processing and A beta42 deposition in a transgenic mouse model of Alzheimer disease. *Proc. Natl. Acad. Sci. U S A.* 94 1550–1555. 10.1073/pnas.94.4.1550 9037091 PMC19829

[B17] Kayed A. Lasagna-Reeve C. A. (2013). Molecular mechanisms of amyloid oligomers toxicity. *J. Alzheimer’s Dis.* 33 S67–S78. 10.3233/JAD-2012-129001IOS 22531422

[B18] Kayed R. Head E. Thompson J. McIntire T. Milton S. Cotman C. (2003). Common structure of soluble amyloid oligomers implies common mechanism of pathogenesis. *Science* 300 486–489. 10.1126/science.1079469 12702875

[B19] Lannfelt L. Möller C. Basun H. Osswald G. Sehlin D. Satlin A. (2014). Perspectives on future Alzheimer therapies: Amyloid-β protofibrils - A new target for immunotherapy with BAN2401 in Alzheimer’s disease. *Alzheimers Res. Ther.* 6:16. 10.1186/alzrt246 25031633 PMC4054967

[B20] Larini L. Shea J. (2012). Role of β-hairpin formation in aggregation: The self-assembly of the amyloid-β(25-35) peptide. *Biophys. J.* 103 576–586. 10.1016/j.bpj.2012.06.027 22947874 PMC3414875

[B21] Lord A. Englund H. Söderberg L. Tucker S. Clausen F. Hillered L. (2009). Amyloid-beta protofibril levels correlate with spatial learning in Arctic Alzheimer’s disease transgenic mice. *FEBS J.* 276 995–1006. 10.1111/j.1742-4658.2008.06836.x 19215301 PMC2752010

[B22] Lorenzen N. Nielsen S. Buell A. Kaspersen J. Arosio P. Vad B. (2014). The role of stable α-synuclein oligomers in the molecular events underlying amyloid formation. *J. Am. Chem. Soc.* 136 3859–3868. 10.1021/ja411577t 24527756

[B23] McDonald J. Cairns N. Taylor-Reinwald L. Holtzman D. Walsh D. (2012). The levels of water-soluble and triton-soluble Aβ are increased in Alzheimer’s disease brain. *Brain Res.* 1450 138–147. 10.1016/j.brainres.2012.02.041 22440675 PMC3319647

[B24] Metzendorf N. Godec A. Petrovic A. Chourlia A. Napoleone A. Syvänen S. (2025). Somatostatin therapy, neprilysin activation, and amyloid beta reduction: A novel approach for Alzheimer’s treatment. *Biomed. Pharmacother.* 189:118325. 10.1016/j.biopha.2025.118325 40633205

[B25] Mrdenovic D. Pieta I. Nowakowski R. Kutner W. Lipkowski J. Pieta P. (2022). Amyloid β interaction with model cell membranes - What are the toxicity-defining properties of amyloid β? *Int. J. Biol. Macromol.* 200 520–531. 10.1016/j.ijbiomac.2022.01.117 35074328

[B26] Nguyen H. Linh H. Krupa P. La Penna G. Li M. (2022). Amyloid β dodecamer disrupts the neuronal membrane more strongly than the mature fibril: Understanding the role of oligomers in neurotoxicity. *J. Phys. Chem. B* 126 3659–3672. 10.1021/acs.jpcb.2c01769 35580354 PMC9150093

[B27] O’Connor T. Sadleir K. Maus E. Velliquette R. Zhao J. Cole S. (2008). Phosphorylation of the translation initiation factor eIF2alpha increases BACE1 levels and promotes amyloidogenesis. *Neuron* 60 988–1009. 10.1016/j.neuron.2008.10.047 19109907 PMC2667382

[B28] Ono K. Tsuji M. (2020). Protofibrils of Amyloid-β are important targets of a disease-modifying approach for Alzheimer’s disease. *Int. J. Mol. Sci.* 21:952. 10.3390/ijms21030952 32023927 PMC7037706

[B29] Peters C. Bascuñán D. Opazo C. Aguayo L. (2016). Differential membrane toxicity of Amyloid-β fragments by pore forming mechanisms. *J. Alzheimers Dis.* 51 689–699. 10.3233/JAD-150896 26890761

[B30] Rissin D. Kan C. Campbell T. Howes S. Fournier D. Song L. (2010). Single-molecule enzyme-linked immunosorbent assay detects serum proteins at subfemtomolar concentrations. *Nat. Biotechnol.* 28 595–599. 10.1038/nbt.1641 20495550 PMC2919230

[B31] Rofo F. Buijs J. Falk R. Honek K. Lannfelt L. Lilja A. (2021). Novel multivalent design of a monoclonal antibody improves binding strength to soluble aggregates of amyloid beta. *Transl. Neurodegener.* 10:38. 10.1186/s40035-021-00258-x 34579778 PMC8477473

[B32] Roher A. Baudry J. Chaney M. Kuo Y. Stine W. Emmerling M. (2000). Oligomerizaiton and fibril asssembly of the amyloid-beta protein. *Biochim. Biophys. Acta* 1502 31–43. 10.1016/s0925-4439(00)00030-2 10899429

[B33] Ruttenberg S. Nowick J. S. A. (2024). turn for the worse: aββ-hairpins in Alzheimer’s disease. *Bioorg. Med. Chem.* 105:117715. 10.1016/j.bmc.2024.117715 38615460 PMC11876106

[B34] Saito T. Matsuba Y. Mihira N. Takano J. Nilsson P. Itohara S. (2014). Single App knock-in mouse models of Alzheimer’s disease. *Nat. Neurosci.* 17 661–663. 10.1038/nn.3697 24728269

[B35] Sandberg A. Luheshi L. Söllvander S. Pereira de Barros T. Macao B. Knowles T. P. (2010). Stabilization of neurotoxic Alzheimer amyloid-beta oligomers by protein engineering. *Proc. Natl. Acad. Sci. U S A.* 107 15595–15600. 10.1073/pnas.1001740107 20713699 PMC2932621

[B36] Sarroukh R. Cerf E. Derclaye S. Dufrêne Y. Goormaghtigh E. Ruysschaert J. (2010). Transformation of amyloid β(1-40) oligomers into fibrils is characterized by a major change in secondary structure. *Cell. Mol. Life Sci.* 68 1429–1438. 10.1007/s00018-010-0529-x 20853129 PMC11114854

[B37] Sengupta U. Nilson A. Kayed R. (2016). The role of amyloid-β oligomers in toxicity, propagation, and immunotherapy. *EBioMedicine* 6 42–49. 10.1016/j.ebiom.2016.03.035 27211547 PMC4856795

[B38] Sevigny J. Chiao P. Bussière T. Weinreb P. Williams L. Maier M. (2016). The antibody aducanumab reduces Aβ plaques in Alzheimer’s disease. *Nature* 537 50–56. 10.1038/nature19323 27582220

[B39] Sims J. Zimmer J. Evans C. Lu M. Ardayfio P. Sparks J. (2023). Donanemab in early symptomatic Alzheimer disease: The TRAILBLAZER-ALZ 2 randomized clinical trial. *JAMA* 330 512–527. 10.1001/jama.2023.13239 37459141 PMC10352931

[B40] Sloane J. Pietropaolo M. Rosene D. Moss M. Peters A. Kemper T. (1997). Lack of correlation between plaque burden and cognition in the aged monkey. *Acta Neuropathol.* 94 471–478. 10.1007/s004010050735 9386780

[B41] Stenh C. Englund H. Lord A. Johansson A. Almeida C. Gellerfors P. (2005). Amyloid-beta oligomers are inefficiently measured by enzyme-linked immunosorbent assay. *Ann. Neurol.* 58 147–150. 10.1002/ana.20524 15984012

[B42] Stern A. Yang Y. Jin S. Yamashita K. Meunier A. Liu W. (2023). Abundant Aβ fibrils in ultracentrifugal supernatants of aqueous extracts from Alzheimer’s disease brains. *Neuron* 111 2012–2020.e4. 10.1016/j.neuron.2023.04.007 37167969 PMC10330525

[B43] Sumner I. Edwards R. Asuni A. Teeling J. (2018). Antibody engineering for optimized immunotherapy in Alzheimer’s disease. *Front. Neurosci.* 12:254. 10.3389/fnins.2018.00254 29740272 PMC5924811

[B44] Tolar M. Hey J. Power A. Abushakra S. (2021). Neurotoxic soluble amyloid oligomers drive Alzheimer’s pathogenesis and represent a clinically validated target for slowing disease progression. *Int. J. Mol. Sci.* 22:6355. 10.3390/ijms22126355 34198582 PMC8231952

[B45] van Dyck C. Swanson C. Aisen P. Bateman R. Chen C. Gee M. (2023). Lecanemab in early Alzheimer’s disease. *N. Engl. J. Med.* 388 9–21. 10.1056/NEJMoa2212948 36449413

[B46] Xiao Y. Ma B. McElheny D. Parthasarathy S. Long F. Hoshi M. (2015). Aβ(1-42) fibril structure illuminates self-recognition and replication of amyloid in Alzheimer’s disease. *Nat. Struct. Mol. Biol.* 22 499–505. 10.1038/nsmb.2991 25938662 PMC4476499

[B47] Xu Y. Shen J. Luo X. Zhu W. Chen K. Ma J. (2005). Conformational transition of amyloid beta-peptide. *Proc. Natl. Acad. Sci. U S A.* 102 5403–5407. 10.1073/pnas.0501218102 15800039 PMC556260

[B48] Yang Y. Zhang W. Murzin A. Schweighauser M. Huang M. Lövestam S. (2023). Cryo-EM structures of amyloid-β filaments with the Arctic mutation (E22G) from human and mouse brains. *Acta Neuropathol.* 145 325–333. 10.1007/s00401-022-02533-1 36611124 PMC9925504

[B49] Yokoyama M. Kobayashi H. Tatsumi L. Tomita T. (2022). Mouse models of Alzheimer’s disease. *Front. Mol. Neurosci.* 15:912995. 10.3389/fnmol.2022.912995 35799899 PMC9254908

[B50] Yu Y. Jans D. Winblad B. Tjernberg L. Schedin-Weiss S. (2018). Neuronal Aβ42 is enriched in small vesicles at the presynaptic side of synapses. *Life Sci. Alliance* 1:e201800028. 10.26508/lsa.201800028 30456353 PMC6238618

[B51] Zielinski M. Peralta Reyes F. Gremer L. Schemmert S. Frieg B. Schäfer L. (2023). Cryo-EM of Aβ fibrils from mouse models find tg-APPArcSwe fibrils resemble those found in patients with sporadic Alzheimer’s disease. *Nat. Neurosci.* 26 2073–2080. 10.1038/s41593-023-01484-4 37973869 PMC10689242

